# Minimization of Parasitic Capacitance between Skin and Ag/AgCl Dry Electrodes

**DOI:** 10.3390/mi15070907

**Published:** 2024-07-12

**Authors:** Sungcheol Hong, Gerard Coté

**Affiliations:** 1 Department of Biomedical Engineering, Texas A&M University, College Station, TX 77843, USA; gcote@tamu.edu; 2 Department of Electrical Engineering, Texas A&M University, College Station, TX 77843, USA; 3 Center for Remote Health Technologies and Systems, Texas A&M Engineering Experiment Station, Texas A&M University, College Station, TX 77843, USA

**Keywords:** bioimpedance, electrocardiogram, Ag/AgCl electrode, skin interface, parasitic capacitor, artery pulse signals, HFSS simulation, comparative analysis

## Abstract

Conventional dry electrodes often yield unstable results due to the presence of parasitic capacitance between the flat electrode surface and the non-uniform skin interface. To address this issue, a gel is typically placed between the electrodes to minimize parasitic capacitance. However, this approach has the drawbacks of being unsuitable for repeated use, limited lifetime due to gel evaporation, and the possibility of developing skin irritation. This is particularly problematic in underserved areas since, due to the cost of disposable wet electrodes, they often sterilize and reuse dry electrodes. In this study, we propose a method to neutralize the effects of parasitic capacitance by attaching high-value capacitors to the electrodes in parallel, specifically when applied to pulse wave monitoring through bioimpedance. Skin capacitance can also be mitigated due to the serial connection, enabling stable reception of arterial pulse signals through bioimpedance circuits. A high-frequency structure simulator (HFSS) was first used to simulate the capacitance when injection currents flow into the arteries through the bioimpedance circuits. We also used the simulation to investigate the effects of add-on capacitors. Lastly, we conducted preliminary comparative analyses between wet electrodes and dry electrodes in vivo with added capacitance values ranging from 100 pF to 1 μF, altering capacitance magnitudes by factors of 100. As a result, we obtained a signal-to-noise ratio (SNR) that was 8.2 dB higher than that of dry electrodes. Performance was also shown to be comparable to wet electrodes, with a reduction of only 0.4 dB using 1 μF. The comparative results demonstrate that the addition of capacitors to the electrodes has the potential to allow for performance similar to that of wet electrodes for bioimpedance pulse rate monitoring and could potentially be used for other applications of dry electrodes.

## 1. Introduction

Electrical potentials called electrophysiological (EP) signals that measure a subject’s condition by attaching diagnostic electrodes to the skin can include electrocardiography (ECG) [1,2,3], electroencephalography (EEG) [4,5,6,7], electromyography (EMG) [8,9], electrooculography (EOG) [10,11] signals, and Bioimpedance (BioZ) [12,13,14,15,16] technologies. Each of these signals can reflect the physiological state of the human body and provide actionable information that can be used by the patient or health care provider. The alternating current flowing through the body via the electrodes can vary in signal quality depending on the quality of the electrodes. Consequently, there is a significant amount of research underway to address this issue and improve signal quality [17,18,19,20,21,22,23,24,25,26].

In the literature, there are primarily three types of electrodes: wet electrodes, dry electrodes, and modified dry electrodes [27,28,29,30,31,32,33]. Dry electrodes are typically composed of an inert conductive material that mechanically couples to the skin for signal transduction. The dry electrodes are often gold-plated or bristle-type and can be of various sizes and shapes. Wet electrodes are the most commonly used and are typically referred to as “wet” since they use a layer of liquid, conductive gel, or paste between the electrode and skin to increase conductivity. The wet electrodes are often made of silver with a coating of silver chloride (Ag/AgCl). Modified dry electrodes can include layers of dry material such as silicon conductive rubber, conductive nanocomposites, or others, including polymer-based screen-printed types.

One of the primary challenges faced by researchers is the presence of parasitic capacitance, which typically ranges from 1 pF to 10 nF [34,35,36] and interferes with precise signal measurement [18,19]. The wet electrode is the most commonly adopted solution to such issues since it contains gel components typically containing many chloride ions, which provides reduced impedance and, thus, improved conduction between the skin–electrode interface [37]. Overall, the wet electrodes allow for very high signal quality, less susceptibility to motion, and hence more stable recordings. However, this solution introduces universal problems of non-reusability and limited lifetime due to gel evaporation. The inability to reuse is particularly problematic in underserved areas because of the cost, and hence, the sterilization and reusability of dry electrodes are more common. Additionally, reports exist of problems such as skin irritation or rash with wet electrodes [38,39]. Further, depending on the clinical application, if the gel needs to be applied, for instance, with EEG caps, then there is the inconvenience of added preparation time, cap cleaning, and hair cleaning. Dry electrodes can thus be advantageous, particularly for EEG, since they have a quicker setup and clean-up time. However, there is often difficulty keeping dry electrodes affixed to the skin; they are more susceptible to motion artifacts and, most importantly, more unstable due to the higher impedance and parasitic capacitance. The modified dry electrodes aim to improve on the disadvantages of dry electrodes; however, these solutions have been primarily focused on reducing resistance and potentially enhancing signal magnitude but did not consider signal quality due to uneven parasitic capacitance [17,40,41,42,43,44].

Our research focuses on the need for a reliable and reusable dry electrode solution that effectively mitigates the effects of parasitic capacitance. Here, we propose a novel approach by incorporating large-value, small-sized capacitors in parallel with the electrodes, effectively minimizing the influence of parasitic capacitance. This approach aims to establish the dominance of the added capacitors over other capacitance sources, rendering them negligible in signal measurement [45]. We validated our proposed solution through high-frequency structure simulator (HFSS) simulations and with a preliminary in vivo measurement of arterial pulse signals from a bioimpedance circuit. This analysis provides insights into the efficacy of our approach in the frequency domain, paving the way for further advancements in dry electrodes for bioimpedance monitoring and potentially physiological monitoring with dry electrodes in general. Our study’s primary contribution lies in the development of a dry electrode system capable of potentially mitigating both parasitic capacitance and capacitance originating from the skin, thus ensuring reusability and reliability. By achieving this objective, we aim to facilitate the adoption of dry electrode technology in various healthcare and research settings, particularly underserved areas where, due to the cost of disposable wet electrodes, they often sterilize and reuse dry electrodes.

## 2. Materials and Methods

### 2.1. Electrical Characteristic Measurements

To measure the circuit’s electrical characteristics, we supplied the power to the circuit with a triple-channel DC power supply (2231A-30-3, Keithley, Solon, OH, USA). Then, we simulated the electrical pulse with a signal generator (AFG1022, Tektronix, Beaverton, OR, USA) and monitored the characteristics of each node with an oscilloscope (MDO3024, Tektronix) [46].

### 2.2. Electromagnetic Field Simulation

Commercial software, Ansys HFSS (Ansys Electromagnetics Suite 2020 R2-HFSS, Ansys, Canonsburg, PA, USA), was used to simulate parameters and distribution of the magnetic and electrical fields from the circuit and tissue. The conductive material was copper, with a finite conductivity of 58 MS m^−1^. The substrate material for the device was set as polyamide with a relative permittivity of 4.3, a dielectric loss tangent of 0.005, and a 5.5 relative per permittivity for the radiation box [47,48]. The radiation region is set as 10% for +X padding, −X padding, +Y padding, −Y padding, and 30% for +Z padding and −Z padding. The power for the excitation port was set as 1 watt. Electrical parameters for arterial wall, skin, fat, and blood were characterized as a function of frequency [49]. All dimensions for the models were taken from reference [50].

### 2.3. Electrical Circuit Simulation

Commercial software OrCAD Capture (PSpice Plugin v16-5-13B, Cadence, San Jose, CA, USA) was used for the electrical circuit simulation. A simulation was run with relative accuracy of the voltages and currents of 0.001. The best accuracy of voltages, currents, and charges were 0.1 µV 1.0 pA, 0.01 pC each. The minimum conductance for any branch was 1.0 × 10^−12^/ohm. DC and bias ’blind’ iteration limits were 150, and DC bias ’best guess’ iteration limit was 20. The transient time point iteration limit was set as 10. For all the simulations, the normal default temperature was 27.0 °C. Auto converges function was used.

### 2.4. Wet Electrodes

Commercial electrodes 3 M Red Dot EKG Snap Electrode (Model number: 408100) were used as the wet electrodes for this study.

### 2.5. Dry Electrodes

Commercial silver/silver chloride (Ag/AgCl) electrodes (Item number: ELT-SA3407) were used as the dry electrodes for this study.

### 2.6. Add-on Ag/AgCl Dry Electrode Fabrication

The add-on electrodes were fabricated by attaching surface-mount device (SMD) capacitors onto Ag-AgCl electrodes (Item #: ELT-SA3407) using commercial product MG Chemicals 8330D-19G 8330D Silver Conductive Epoxy Adhesive, 19 g kit.

The SMD capacitors used were as follows: 100 pF: Multilayer Ceramic Capacitors MLCC—SMD/SMT 100 pF+/−5% 25 V C0G 0 0201 (CL03C101JA3NNNC, Samsung Electro-Mechanics, Suwon, Republic of Korea); 10 nF: Multilayer Ceramic Capacitors MLCC—SMD/SMT 10 nF+/−10% 25 V X5R 0 0201 (CL03A103KA3NNNC, Samsung Electro-Mechanics); 1 μF: Multilayer Ceramic Capacitors MLCC—SMD/SMT 1 μF+/−20% 16 V X5R 06 0201 (CL03A105MO3NRNC, Samsung Electro-Mechanics).

### 2.7. Electrical Components

The electrical components used in the circuit for this study include the microcontroller unit (NRF52832, Nordic Semiconductor, Trondheim, Norway), operational amplifier (OPA2387, Texas Instruments, Dallas, TX, USA), instrumentation amplifier (INA823, Texas Instruments), and modulator/demodulator (AD630, Analog Devices, Wilmington, MA, USA).

### 2.8. Informed Consent

Informed consent was obtained from all individuals included in this study.

### 2.9. Ethical Approval

The research related to humans has been performed with the approval of the Institutional Review Board of Texas A&M University (IRB number: IRB2022-0227).

## 3. Results

### 3.1. Equivalent Circuit Analysis of the Skin–Electrode Interface

**Figure 1:**
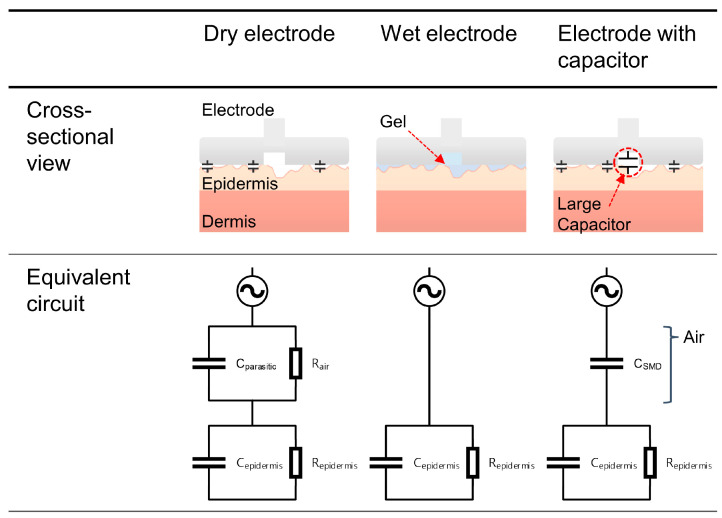
Comparison between the conventional dry electrode, wet electrode, and the proposed method.

Figure 1 illustrates an equivalent circuit analysis between our proposed electrode-with-capacitor approach and the conventional Ag/AgCl dry electrode and wet electrode. In traditional dry electrodes, a gap between the electrode and the uneven skin surface results in capacitance components that affect the signal pathway, leading to decreased signal reliability. To address this issue, wet electrodes have been employed to eliminate the gap between the electrode and the skin surface by filling it with a conductive layer of gel. Our solution addresses the problem by connecting high-capacitance SMD capacitors in parallel to the dimple part of the dry electrode, sized at EIA 0201 (0.024 in × 0.012 in or 0.6 × 0.3 mm) [51,52]. The reason for using the small size of EIA 0201 is to mitigate the possibility of introducing additional unwanted capacitance between the surface area of the capacitor and the skin when attaching larger capacitors. As shown in Figure 1, conventional dry electrodes exhibit unwanted and unpredictable impedance values due to the gap between the electrode and the skin. As depicted in the figure, wet electrodes are represented by a simplified equivalent circuit in which, by applying the gel, effectively shows these added impedance components are removed [53,54,55,56,57,58]. In our solution, intentionally connecting capacitors of larger values in parallel does not entirely eliminate the components but rather represents a simplified circuit that enables us to overcome the effects of the parasitic capacitance values and resistance resulting from the gap.

**Figure 2:**
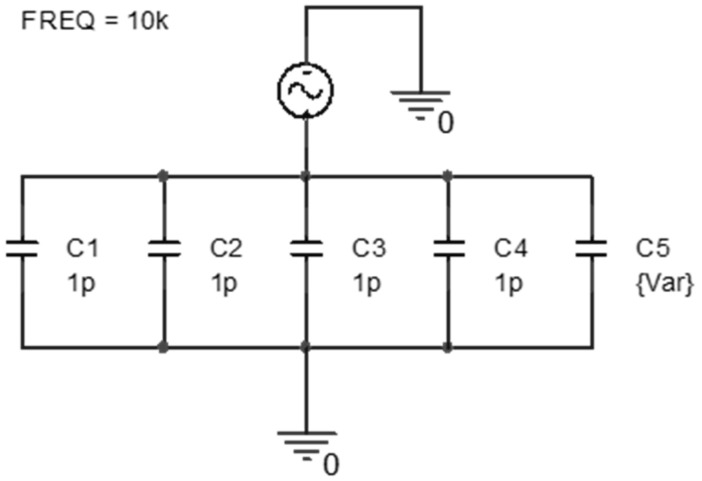
Simulation setup by placing larger capacitors in parallel to the parasitic capacitance, controlling the total impedance.

**Figure 3:**
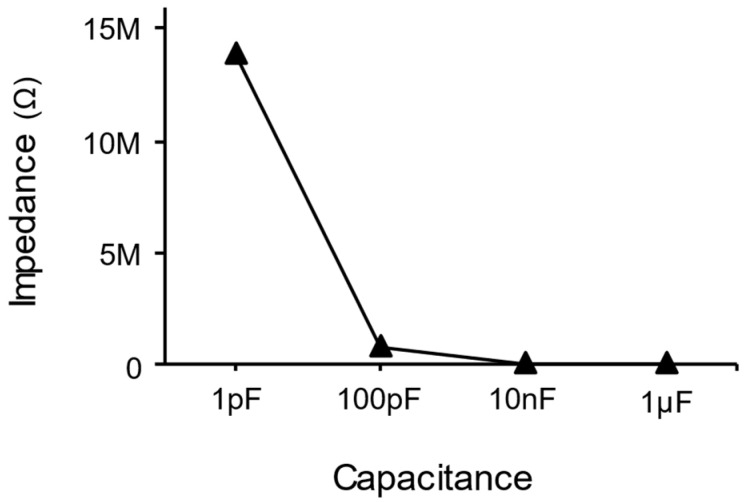
The graph of the total impedance converging as the value of the add-on capacitor increases. Note that 1 pF corresponds to 13.91 MΩ, 100 pF corresponds to 668.57 kΩ, 10 nF corresponds to 6.95 kΩ, and 1 μF corresponds to 70 Ω.

This is because intentionally placing capacitors larger than the parasitic capacitance in parallel ensures that the capacitance remaining between the entire skin and electrode converges to the value of the added capacitor, becoming controllable. Since the added capacitor dominates the total sum, variations in the parasitic capacitance have minimal impact on the overall circuit perspective, resulting in a more stable outcome. In Figure 2, to demonstrate this, the impedance values were simulated by sweeping the value of one capacitance connected in parallel with four other small-value capacitors. As shown in Figure 3, when the value of the capacitor connected in parallel is sufficiently large, the overall impedance converges to a single value. The range of impedance values varies widely with the capacitance, namely for Figure 3; 1 pF corresponds to 13.91 MΩ, 100 pF corresponds to 668.57 kΩ, 10 nF corresponds to 6.95 kΩ, and 1 μF corresponds to 70 Ω.

This can also be mathematically expressed as follows:

(1) $$C_{total}=C_{1}∥C_{2}∥C_{3}∥C_{4}∥C_{add-on}$$

(2) $$C_{total}=C_{1}+C_{2}+C_{3}+C_{4}+C_{add-on}$$

(3) $$\left(where,\;C_{add-on}\gg C_{1}+C_{2}+C_{3}+C_{4}\right)$$

(4) $$C_{total}≅C_{add-on}$$

Since the impedance of the gap between the electrode and skin converges to the value of the added capacitor, this now becomes a controllable parameter. Furthermore, since the capacitance value is sufficiently large, the impedance due to the gap itself converges to a small value according to the following equation:

(5) $$X_{C}=\frac{1}{j\omega C_{add-on}}$$

### 3.2. HFSS Simulation

**Figure 4:**
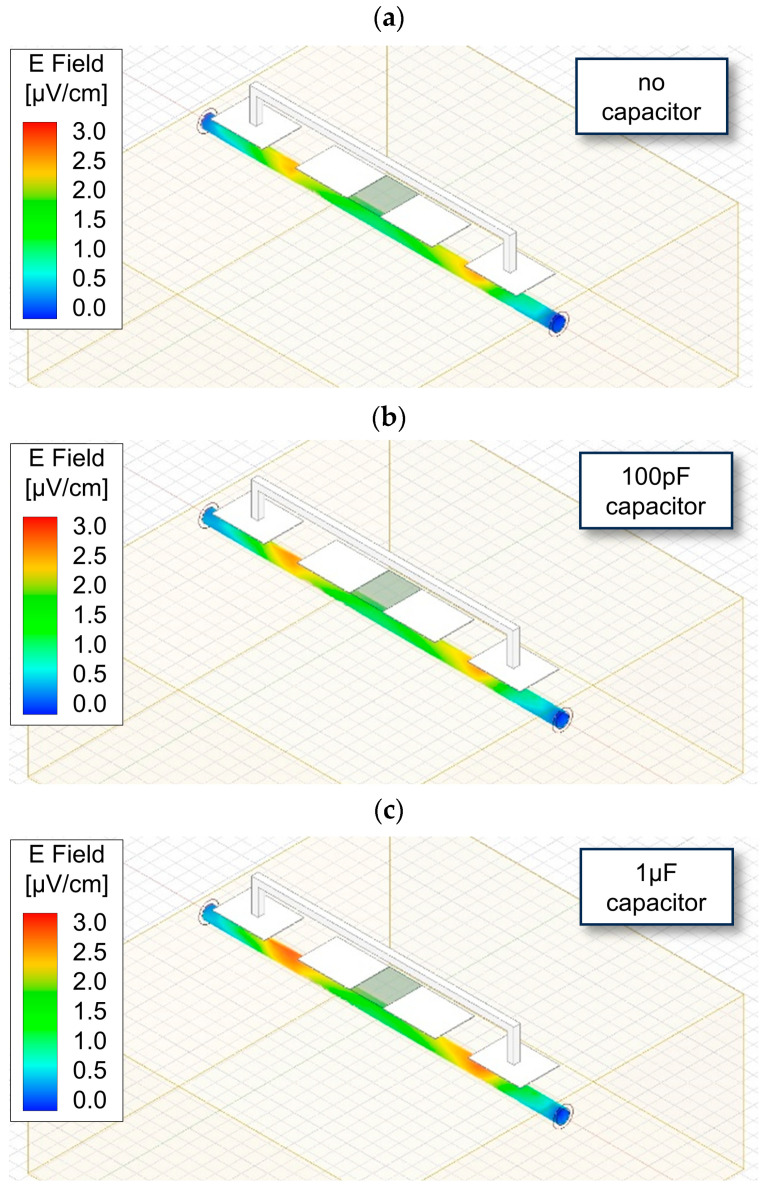
To observe the influence of the add-on capacitor, the setup and results of HFSS simulations are shown: (**a**) without an add-on capacitor; (**b**) with a 100 pF add-on capacitor; (**c**) with a 1 μF add-on capacitor.

**Figure 5:**
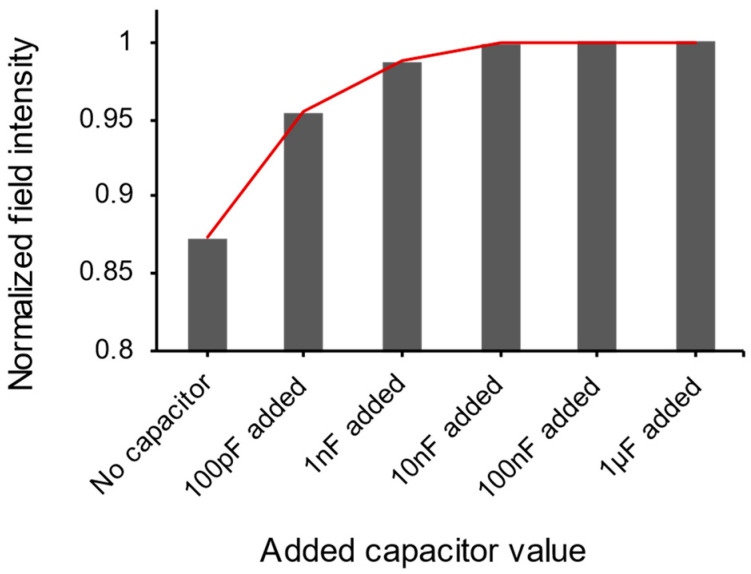
The graph depicts increasing changes in the field intensity as a function of increasing the add-on capacitance between the actual skin and electrode.

In this study, HFSS simulations were initially utilized to investigate the interface between the bioimpedance circuit and the skin [59,60,61]. Figure 4 shows the HFSS simulation setup and illustrates a reduction in the effect of the parasitic capacitance arising from uneven skin when the add-on capacitor attached to the electrode can be observed [52]. In the first image of Figure 4, the electric field (E-field) flowing along the blood vessels is depicted without the add-on capacitor on the uneven skin surface and shows the E-field from the electrode down the blood vessel is relatively weak, whereas, from the second and third images of Figure 4, the E-field flowing through the blood vessels becomes stronger as the capacitance increases. To further refine this observation, an increase in the add-on capacitance values was performed, and the results regarding the field intensity were observed, as presented in Figure 5. These results show that the normalized field intensity is 0.873 without the capacitor and, with increases in the add-on capacitor, the detected field intensity increases sequentially to 0.954, 0.987, 0.998, 1, and 1. This indicates that the add-on capacitor impacts the mitigation of parasitic capacitance. However, it is notable that beyond a certain threshold, the effectiveness of this mitigation is maximized.

### 3.3. Capacitor Add-on Ag/AgCl Dry Electrode

**Figure 6:**
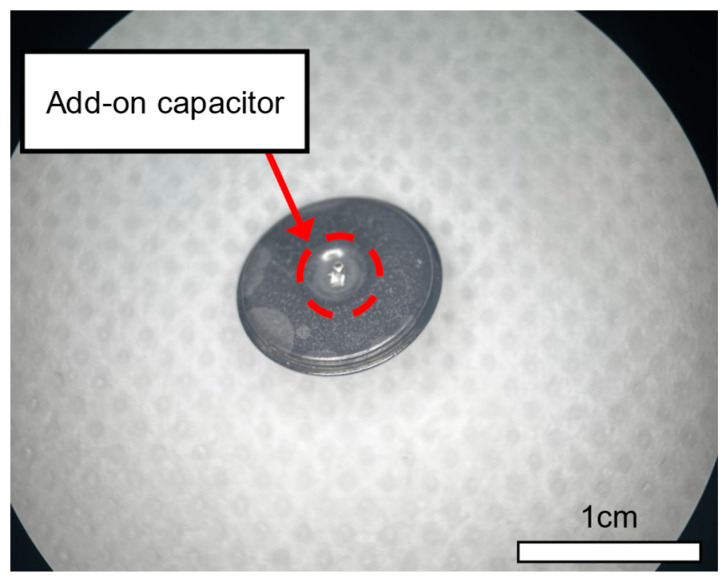
Photograph of the add-on capacitor electrode used in the experiment, which was applied with adhesive silver epoxy.

Figure 6 depicts an actual image of the add-on capacitor electrode used in the experiment. Adhesive silver epoxy was applied to one side of the SMD capacitor, which was then connected to the dimple portion of a commercial dry electrode for use in the experiment. In this way, both the contact surface of the SMD and the electrode make contact with the skin, creating a configuration where the parasitic capacitance and SMD capacitance are positioned in parallel. Additionally, using the small size of EIA 0201 for the SMD capacitors minimizes the surface area of the SMD and reduces the occurrence of additional parasitic capacitance between the SMD and the skin.

### 3.4. Bioimpedance Artery Pulse Monitoring Circuit

**Figure 7:**
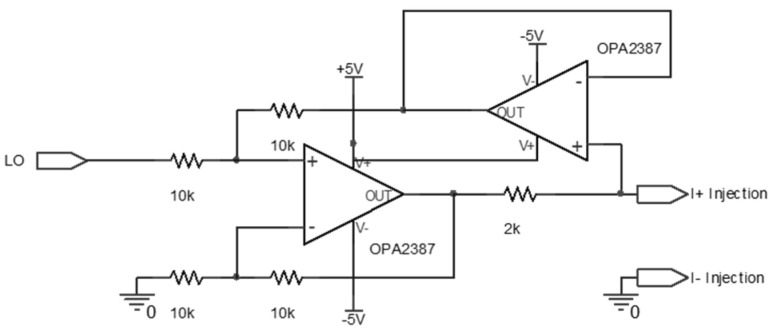
Schematic diagram of the Howland current pump circuit used in the study.

**Figure 8:**
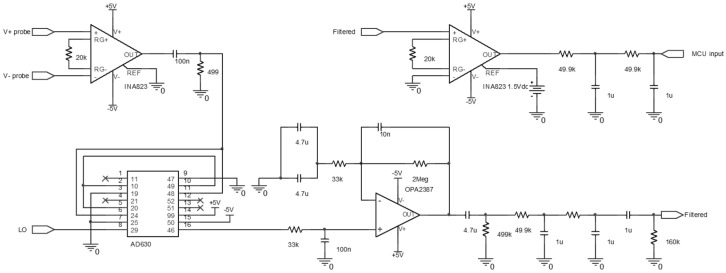
The circuit diagram for bioimpedance signal processing following the detection probe used in the experiment.

Figure 7 and Figure 8 depict the bioimpedance circuit used to test the electrodes. A sinusoidal waveform generated by the microcontroller unit (MCU)’s pulse with modulation (PWM) is filtered to match 10 kHz [52,62,63,64], and then the current pump in Figure 7 is designed to supply a constant current regardless of the load’s impedance value [65,66,67].

After the current injection, the voltage detected by the detection probe passes through the circuit, as shown in Figure 8. The circuit passes through an instrumentation amplifier (INA) and then through a demodulation integrated circuit (IC). In the demodulation IC, the same method is employed for demodulating amplitude modulation (AM) signals. By multiplying the detected signal by the carrier frequency, which is the same as the injected frequency, the signal is separated into two components: one at twice the injected frequency and the other at 0 Hz [68,69]. Subsequently, the signal goes through low-pass and band-pass filters, and only the original signal is recovered, which is then recorded on the MCU analog input pin [70,71]. The same circuit was applied to all electrodes for this study.

### 3.5. Comparative Analysis of the Signals

**Figure 9:**
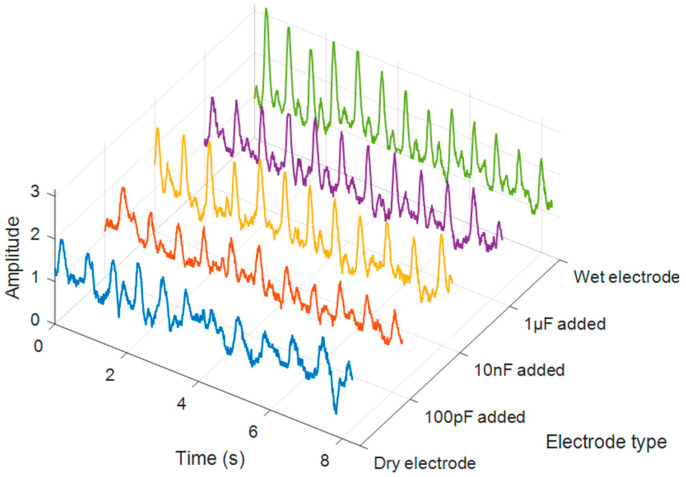
Time-domain bioimpedance signals measured using various electrodes.

The obtained signals are shown in Figure 9. The experiment compared dry electrodes, electrodes with 100 pF capacitors, electrodes with 10 nF capacitors, electrodes with 1 μF capacitors, and wet electrodes in a single subject. As seen in the figure, there were no significant issues in measuring the pulse rate in all experiments. Regardless of the type of electrode, the heartbeats per minute (BPM) ranged from 75 to 95, within the normal range [72,73,74]. The in vivo systolic amplitude varied with both time and across electrodes; however, the waveform distortion was much higher with dry electrodes and with those with small capacitance.

**Figure 10:**
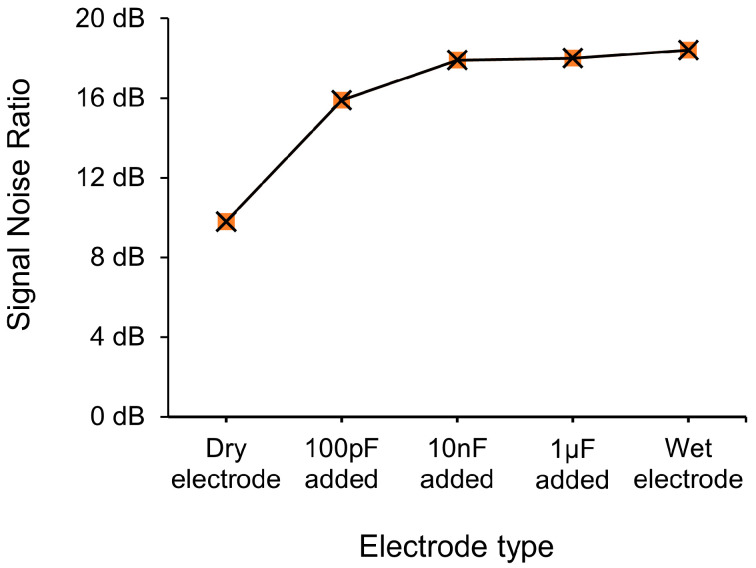
Comparison of spurious SNR measured using various electrodes.

For a more accurate comparison, the spurious signal-to-noise ratio (SNR) was compared in Figure 10 and Table 1, which measures the ratio of desired signal power to unwanted spurious signal power in a system [75]. It was observed that the SNR values increased sequentially from dry electrodes to electrodes with 100 pF, 10 nF, and 1 μF capacitors and to wet electrodes, with values of 9.8 dB, 15.9 dB, 17.9 dB, 18 dB, and 18.4 dB, respectively. This indicates that the addition of a capacitor and its value can improve the signal quality to a level comparable to that of wet electrodes. In particular, the difference between dry electrodes and 1 μF capacitors was 8.2 dB, indicating a significant improvement in signal quality by approximately 6.6 times.

## 4. Conclusions

In this study, we investigated a simple method to address the irregular parasitic capacitance between the skin and dry electrodes during bioimpedance measurements. Specifically, we minimized the parasitic capacitance by using a large capacitance to the dry electrode, which is effectively in parallel. When sweeping capacitance values, in the HSSF simulation, the normalized field intensity values without the capacitor were only 0.87, compared to 0.95 at 100 pF, 0.98 at 1 nF, and reaching a maximum of 1 at 10 nF and higher. Furthermore, when using the proposed dry electrode with the added capacitor method, the SNR of the dry electrode alone was only 9.8 dB, whereas with increasing capacitance values, it increased to 15.9 dB, 17.9 dB, and ultimately up to 18 dB, similar to the 18.4 dB level of the wet electrode. This approach shows the ability of the dry electrodes with proper capacitance to achieve comparable SNR values of wet electrodes and thus have the potential to be used to resolve problems associated with wet electrodes, including allowing for electrode reusability, providing a solution that is longer lasting, and avoiding the potential for skin irritation since there is no need for the gel. In future research, other EP applications could be tested (e.g., ECG, EEG, EOG, etc.), more subjects need to be added, and a more consistent fabrication process needs to be considered that could lead to the mass production and uniform quality of dry electrodes with built-in capacitors.

## Figures and Tables

**Table 1:** Quantitative details for SNR.

Electrode Type	1st Harmonic	Spurious Noise	SNR
Dry electrode	−14 dB	−23.8 dB	9.8 dB
100 pF added	−16.3 dB	−32.2 dB	15.9 dB
10 nF added	−10.4 dB	−28.3 dB	17.9 dB
1 μF added	−12.5 dB	−30.5 dB	18.0 dB
Wet electrode	−11.2 dB	−29.6 dB	18.4 dB

## Data Availability

The original contributions presented in the study are included in the article, further inquiries can be directed to the corresponding author.
